# Adapting Schools to Climate Change with Green, Blue, and Grey Measures in Barcelona: Study Protocol of a Mixed-Method Evaluation

**DOI:** 10.1007/s11524-023-00814-y

**Published:** 2024-01-18

**Authors:** Marta Sanz-Mas, Mònica Ubalde-López, Sílvia Borràs, Sílvia Brugueras, Xavier Continente, Carolyn Daher, Marc Marí-Dell’Olmo, María José López

**Affiliations:** 1 Agència de Salut Pública de Barcelona (ASPB), Pl. Lesseps 1, 08023 Barcelona, Spain; 2 Departament de Ciències Experimentals i de La Salut (DCEXS), Universitat Pompeu Fabra, Doctor Aiguader 88, 08003 Barcelona, Spain; 3 ISGlobal, Barcelona Institute for Global Health, Doctor Aiguader 88, 08003 Barcelona, Spain; 4 Consortium for Biomedical Research in Epidemiology and Public Health (CIBERESP), Av. Monforte de Lemos 3-5, Pabellón 11, Planta 0, 28029 Madrid, Spain; 5 Institut de Recerca Sant Pau (IR SANT PAU), Sant Quintí 77-79, 08041 Barcelona, Spain

**Keywords:** Climate change, Urban planning, Nature-based solutions, Urban health, Children, Schools, Community participation, Program evaluation, Mixed methods, Social innovation

## Abstract

Under the framework of the Urban Innovative Actions program of the European Commission, in 2020, 11 primary schools in Barcelona were transformed into climate shelters by implementing green, blue, and grey measures. Schoolyards were also opened to the local community to be used during non-school periods. Here we present the study protocol of a mixed-method approach to evaluate the effectiveness of the interventions in terms of improving environmental quality and health for users. We evaluated school level through the following: (1) quantitative pre-post quasi-experimental study, and (2) qualitative evaluation. The quantitative study included measures of (a) environmental variables (collected via low-cost and non-low-cost sensors), (b) students’ health and well-being (collected via health questionnaires, attention levels test, and systematic observations), and (c) teachers’ health and well-being (collected via thermal comfort measurements and health questionnaires). The qualitative methods evaluated the perceptions about the effects of the interventions among students (using Photovoice) and teachers (through focus groups). The impact of the interventions was assessed at community level during summer non-school periods through a spontaneous ethnographic approach. Data collection started in August 2019 and ended in July 2022. The evaluation provides the opportunity to identify those solutions that worked and those that need to be improved for future experiences, as well as improve the evaluation methodology and replication for these kinds of interventions.

**Supplementary Information:**

The online version contains supplementary material available at 10.1007/s11524-023-00814-y.

## Introduction

### Climate Change, Urban Planning, and Health

Climate projections indicate increases in heat wave frequency, duration, and amplitude due to global warming [1]. Extreme heat has been associated with premature mortality and morbidity due to cardiovascular, respiratory, and psychiatric conditions [2–4].

Urban populations are highly exposed to heat due to the Urban Heat Island (UHI) [5], as well as to high levels of air pollution and lack of green spaces [6]. In Barcelona, for instance, 363 deaths could be attributed to UHIs in 2015 during normal summer temperatures [7]. Further, 20% of premature mortality in Barcelona is attributable to urban environmental exposures that do not meet the recommendations of international guidelines in terms of heat, air pollution, and access to green spaces, among others [8].

Such environmental exposures can be mitigated by urban planning interventions. Providing access to green areas and blue spaces (i.e. outdoor environments that prominently feature water and are accessible to humans [9]) can provide significant benefits for physical and mental health by reducing air and noise pollution and heat, and providing places for physical activity, leisure, and social interaction [10–12]. Shade infrastructure and building retrofit measures are also beneficial for health by reducing the risk of heat stress [13, 14].

### “Climate Shelters in Schools” in Barcelona

School adaptation to climate change can contribute to the creation of climate-resilient cities. Children are especially vulnerable to high temperatures [14]. Exposure to natural and shaded environments has been positively associated with their cognitive, physical, and social well-being [14–17].

The pilot project “Climate shelters in schools” was conducted in Barcelona from 2018 to 2022, supported by the 3rd call of the Urban Innovative Actions program of the European Commission. The project aimed to transform 11 primary schools (across all city districts) into climate shelters by improving thermal comfort in the school infrastructure while creating healthier, more playful, and inclusive schoolyards. A series of measures were proposed: GREEN (planting trees, adding green walls with climbing plants, and creating gardens with Mediterranean species while replacing hard surfaces in schoolyards with more natural elements), BLUE (inclusion of fountains for drinking, playing, and cooling purposes), and GREY (installing pergolas, canopies, and seating areas in schoolyards, as well as improving thermal conditions of the school buildings with internal and external shade devices, roof insulation, and natural cross-ventilation). A different mix of interventions was implemented depending on the needs of each specific school. Overall, a total of 1000 square meters of concrete were replaced with natural components, 2213 square meters of shade were generated using pergolas and canopies, 74 trees were planted, and 26 new water sources were installed [18].

This innovative project also included a pedagogical dimension (i.e. classroom activities and workshops on climate change-related topics) and a participatory design process, where school communities collaborated with the technical and scientific partners to choose the most appropriate interventions and monitor and evaluate results.

Schoolyards were opened to neighbourhoods as climate shelters during non-school periods through the program “Patis escolars oberts al barri” [19] (“Neighbourhood Open Schoolyards”) to extend the benefits to the surrounding communities. A significant emphasis was given to the monitoring and evaluation of the project process and interventions to demonstrate impacts and understand replicability of the climate shelters model.

### Conceptual Framework of the “Climate Shelters in Schools”

**Fig. 1:**
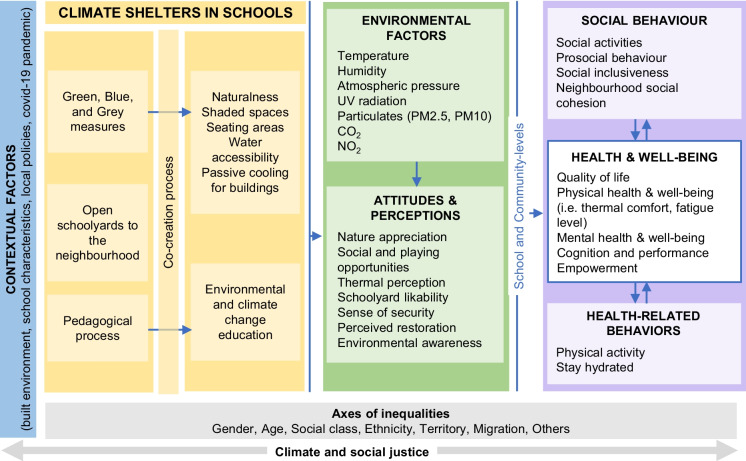
Conceptual framework of the potential effects of the interventions (own elaboration)

As shown in Fig. 1, we created a conceptual framework for the evaluation of the “Climate shelters in schools”. We described the direct and indirect potential impacts of the intervention on health and well-being, considering intermediate and contextual factors that could mediate such effects. This framework was developed based on the evidence found in the literature regarding the effects of climate change and urbanisation on health [3, 6, 14], the impact of similar type of interventions on health and well-being [17, 20–24] and previous published conceptual models [25–28]. According to our framework, green, blue, and grey interventions implemented as part of the “Climate shelters in schools” program are expected to have a direct, positive impact on school’s environmental conditions (especially temperature) and air quality, as well as on users’ attitudes and perceptions towards the school environment (e.g. nature appreciation, thermal perception, schoolyard likability). Pedagogical activities could involve the students as co-creators in the project and enhance their climate awareness.

Subsequently, the interventions could also lead to co-benefits as an improvement of physical and psychological well-being (including thermal comfort), cognitive aspects, health-related behaviours (e.g. physical activity), and social interactions among users (e.g. social inclusiveness and social cohesion). Such effects could be mediated by contextual and intermediate factors, such as socioeconomic and demographic profiles of the neighbourhood, gender, or age.

The impact of COVID-19 pandemic (i.e. school closures, limitation of the schoolyard use and social interaction) on both the implementation and the effect of the interventions need to be considered. The distribution of transformed schools across all districts ensures more equitable benefits, contributing to climate and social justice.

### Objectives

This protocol describes the evaluation strategy designed to assess the impact of the implemented measures in schools.

The general objective of the study is to evaluate the effects of the green, blue, and grey interventions in 11 primary schools in Barcelona on the environment and health, at school and community levels.

Specifically, we seek to evaluate the impact of the interventions on environmental conditions (including temperature and humidity) and air quality (NO_2_ and particulate matter—PM). We aim to determine the effect of the intervention on students’ perceptions of the school environment, health and well-being, attention levels, and climate awareness. We plan to assess the impact of the interventions on students’ use of the schoolyard areas, physical activity, and social behaviour during recess time. We also aim to evaluate the effect of the interventions on teachers’ perceptions of thermal comfort and school environmental quality. Finally, we seek to assess the use of open schoolyards as climate shelters by the local community members and their perceptions of thermal comfort and well-being.

## Materials and Methods

### Study Design

We followed a mixed-method evaluation approach based on a triangulation design model that included an impact evaluation at school and community levels. To assess the effect of the interventions at school level, we used a quantitative pre-post quasi-experimental design to compare impacts on school’s environmental and air quality parameters, as well as on students and teachers’ health and well-being, among the intervention (IG) and comparison (CG) groups before and after the interventions. We also included a qualitative evaluation of student and teacher’s perceptions about the effects of the interventions. For the community-level assessment, we designed a qualitative evaluation among local community members that used the schoolyards as a climate shelter.

### Study Population

The study population included 11- to 12-year-old students from the 6th grade (as classified by the Spanish educational system) and teachers from 21 primary public schools in Barcelona (Spain) during the academic year 2020–2021, as well as residents or visitors that use the schoolyards during non-school hours.

The intervention group (IG) included the 11 public primary schools from the city of Barcelona selected to receive the intervention. A vulnerability score was created and used to determine eligibility for the intervention among those schools that had applied to participate in the project. Ensuring representativeness of all city districts, those public primary schools with higher score were the ones selected to receive the intervention.

Vulnerability score resulted from the combination of 4 different dimensions: impact, environment, buildings, and schoolyard. Regarding impact dimension, schools that had two or more lines, had summer camps at their own facilities, and shared either the playground or the building with a nursery or high school, received higher scores. For environment dimension, schools receiving higher scores were those located in areas of maximum vulnerability to heat waves (above average) based on Barcelona Climate Plan 2018–2030, zones of maximum air pollution (> 60 µg/m^3^ of nitrogen dioxide), and areas with lower presence of green (compared to average) according to the Normalized Difference Vegetation Index Map of Barcelona. For buildings dimension, schools that received higher scores were those with more than 17 classrooms receiving direct sun for more than 2 h (higher to the average), with more than 9 classrooms directly beneath the roof of the building (higher to the average), with a C, D, E, or F energy efficiency certificate and/or with less than 3 heat protection systems. Regarding schoolyard dimension, schools that had more than 75% of the schoolyard with hard surface or receiving direct sunlight, and schools that had no presence of water points, received higher scores.

If schools from the same district received the same ranking score during the selection process, the criterion of school complexity was applied. This was defined on the basis of the families’ level of studies, employment situation, and income, as well as the special needs of the students. Finally, to be eligible for the intervention, schools were required to become part of the “Patis escolars oberts al barri” program [19], opening their schoolyard to the neighbourhood during non-school periods. Further details on selection process are described elsewhere [29].

The comparison group (CG) included 10 public primary schools with similar characteristics to the IG that were not selected for intervention. Eligibility criteria were as follows: (1) having the same score as the intervention school on at least half of the eligible criteria for 2 or more scoring sections (impact, environment, building, schoolyard), and (2) belonging to the same district as the school from the IG, whenever possible; otherwise, schools with similar IG scores were selected from other nearby neighbourhoods.

### Measurement Procedure and Outcome Measures

**Fig. 2:**
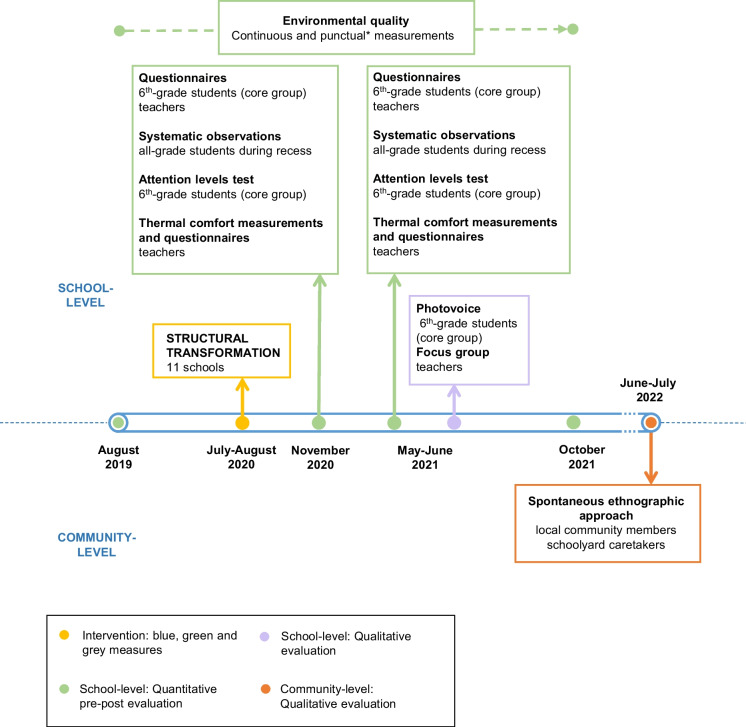
Data collection timeline. *NO_2_ measurement campaigns (August 2019, January 2020, July 2020, January 2021 and July 2021); periodic additional temperature measurements (June–July 2020, and June–July 2021). (Own elaboration)

Different subsamples, methodologies, and techniques used to evaluate the effect of the interventions on multiple environmental and health outcomes are described below and summarised in Table 1. Structural transformation of the schoolyards was carried out between July and August 2020. Data collection started in August 2019 and ended in July 2022. Figure 2 shows the timeline of the evaluation process. Schools were closed before and during intervention’s implementation due to COVID-19 pandemic. Therefore, pre-intervention questionnaire data were obtained retrospectively after the intervention (November 2020), which will allow us to estimate pre-post changes among the different outcomes. This situation did not affect the environmental measures which continued across the school’s closure. Some monitoring measures were not suitable for retroactive pre-testing (i.e. attention level tests, systematic observations of the schoolyard, measured thermal comfort using sensors) and, thus, were assessed following a post-post strategy, in November 2020 and May 2021, to allow evaluating the potential effects of the interventions during the school year after the transformations performed during summer 2020.

**Table 1:** Study design of the mixed method evaluation: methodology, main dimensions, study sample, information source and measures

Methodology	Main dimensions		Schools involved	Participants	Source	Measures
Quantitative quasi-experimental evaluation	Environmental conditions and air quality	Temperature and other environmental variables	11 schools (IG)	N/A	Low-cost sensors (Smart Citizen Kits)	Pre and post (continuously)
		Nitrogen dioxide	11 schools (IG)	N/A	Passive diffusion tubes	3 pre, 2 post
	Health and well-being (students)	Perception of school environment, health and well-being, schoolyard use, social behaviour, climate awareness	21 schools (11 IG, 10 GC)	851 6th-grade students	Questionnaires	2 post^a^
		Attention levels	8 schools (4 IG, 4 GC)	302 6th-grade students	ANT test	2 post
		Schoolyard use, physical activity, social behaviour	11 schools (IG)	All grade students in each school during recess	Systematic observations	2 post
	Health and well-being (teachers)	Measured thermal comfort	8 schools (4 IG, 4 GC)	80 teachers	Individual portable sensors (iButton™)	2 post
		Perceptions on thermal comfort	8 schools (4 IG, 4 GC)	80 teachers	ASHRAE questionnaire	2 post
		Perceptions on school environmental quality	21 schools (11 IG, 10 GC)	151 teachers	Questionnaires	2 post
Qualitative evaluation	Student perceptions on the intervention’s impact		4 schools (IG)	59 6th-grade students	Photovoice	1 post
	Teacher perceptions on the intervention’s impact		11 schools (IG)	11 teachers	Focus groups	1 post
	User attitudes, experiences, and perceptions of climate shelters among local community		11 open schoolyards (IG)	76 visitors and 18 caretakers of climate shelters	Spontaneous ethnographic approach	1 post

*IG* intervention group, *CG* comparison group, *N/A* not applicable. ^a^Includes retrospective questions for pre-intervention indicators

#### Quantitative Pre-post Quasi-Experimental Evaluation

##### Environmental Conditions and Air Quality

To evaluate the impact of the intervention on environmental conditions, each of the 11 IG schools was equipped with six Smart Citizen Kits (SCKs) v2.1, deployed both in outdoor and indoor locations, and both in intervention and reference areas (control kits). These low-cost sensor kits measured a range of parameters including air temperature, relative humidity, barometric pressure, particulate matter (PM1, PM2.5, and PM10), total volatile organic compounds (tVOC), ambient light, and noise level. Information regarding the performance of the sensors included in the SCKs can be found elsewhere [30]. Data were collected every 5 min from August 2019 to October 2021. Furthermore, we conducted a 1-day measurement campaigns in each schoolyard using 7 to 12 additional SCKs before (summer 2020) and after the interventions (summer 2021). Data were collected every 1 min, with sensor kits distributed across the schoolyard to attain a higher temporal and spatial resolution in temperature measurements.

To assess the impact of the intervention on school’s air quality, NO_2_ levels were measured using Palmes-type diffusion tubes, consisting of small acrylic tubes containing a chemical reagent (triethanolamine) to absorb the pollutant directly from the air. Five measurement campaigns were simultaneously performed in the intervention schools in August 2019, January and July 2020, and January and July 2021. For each campaign, four NO_2_ diffusion tubes were placed in each school: one outside (at the school’s main entrance), two in the schoolyard, and one indoors (in a classroom). Tubes were placed at typical human breathing height, that is, approximately 1.5 to 2.0 m above ground level for 3 weeks [31–33]. Concentrations of nitrite ions, and hence NO_2_, chemically absorbed, were quantitatively determined by UV/Visible spectrophotometry.

##### Health and Well-Being of Students

We evaluated the effect of the intervention on student’s perceptions of the schoolyard, health and well-being, attention levels, and climate awareness, as well as their use of the schoolyard, physical activity, and social behaviour.

First, we administered online questionnaires to a subsample of 851 6th-grade students (11–12 years old), from the 11 IG and 10 CG schools (447 and 404 children, respectively). IG participants were those students who participated in the co-creation process of the selected interventions during the previous academic year 2019–2020 (core group). Questionnaires were administered to IG and CG at two different periods after the intervention (November 2020 and May 2021). We included retrospective questions in the first assessment to obtain pre-intervention data.

Questionnaires were based on selected items from validated tools. First, we gathered indicators related to participants’ sociodemographic data. We added questions regarding students’ characteristics, including sex (boys or girls), age (date of birth), and place of birth. We included items related to students’ family characteristics, such as the family affluence scale [34] and the family structure (following the Health Behaviour in School-aged Children—HBSC—study protocol [35]). We also asked about parents’ place of birth and their levels of studies. Finally, we collected information about school’s neighbourhood socioeconomic position (low, medium, high) on the basis of data from the territorial distribution of family income per capita in Barcelona in 2017 [36].

We assessed how much the students liked their schoolyard using a 1-to-10 scale [17]. Students’ affinity for the schoolyard was assessed by including the three-item tool (1-to-5 scale) developed by Collado S. [21]. We evaluated student’s perceptions of green, shaded and seating areas, and possibility to do activities in the schoolyard (very poor, poor, acceptable, good, or very good) based on MM 060 School questionnaire [37]. We also selected a series of questions from the questionnaire “Play Activities in the Schoolyard” related to schoolyard use: type of activities, level of physical activity, and social behaviour during recess (social play, mixed-gendered play) [17]. The KIDSCREEN-10 [38] was used to evaluate students’ self-perceived health and quality of life. Physical, psychological, and school well-being were evaluated using KIDSCREEN-27 dimensions: physical well-being, psychological well-being, and school environment [38]. MM 060 School questionnaire [37] was used to evaluate perceptions on indoor school environment conditions (e.g. indoor temperature). Climate change awareness was evaluated through The Climate Change Attitude Survey [39] and Pro-environmental behaviour scale [21].

Second, we evaluated the impact on students’ attention levels by selecting a subsample of 302 (145 IG and 157 CG) 6th-grade students (core group), from a sample of 8 schools (4 IG and 4 CG). The schools were selected according to diversity criteria (e.g. socioeconomic status, location in the neighbourhood, school size, greenness). Children completed the Network Flanker Task (ANT) attention level test (previously piloted) at two time points after the implementation of interventions, November 2020 and May 2021.

The ANT instrument is a validated cognitive test, consisting of 128 sequences (lasts about 10 min), that measures alertness, orientation, and executive attention [40]. Children had to select on a computer keyboard, as quickly as possible, the orientation (left or right) of the central arrow in a row of five arrows. Indoor environmental quality during the performance of the ANT test was evaluated (i.e. temperature, relative humidity, CO_2_, PM2.5, and black carbon) using different monitoring instruments.

Finally, we also assessed the effect of the intervention on students’ use of the schoolyard, physical activity, and social behaviour by performing systematic observations of the use of schoolyard space during recess in two periods after intervention implementation (November 2020 and May 2021). We observed all children, from preschool to 6th grade (3 to 11 years old), during recess in the schoolyard in the 11 IG schools.

**Fig. 3:**
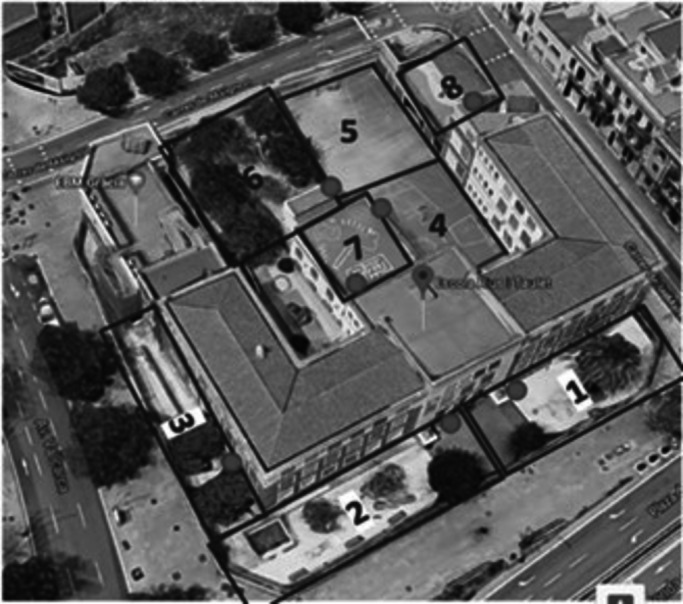
Example of target areas and observation points

The observations were carried out using the SOOPEN (System for Observing Outdoor Play Environments in Neighbourhood Schools) tool, a tailored systematic observation tool designed within the evaluation study to observe the recess time in the playground at the group-level interaction. SOOPEN is based on two validated and widely used tools: SOPLAY (System for Observation of Play and Leisure Activity in Youth [41]) and SOCARP (System for observing children’s activity and relationships during play [42]). SOOPEN incorporates in a single tool the observation of children’s group-level dynamics, considering gender, social interaction, physical activity levels, and type of activities. Contextual information was also collected, such as the grade of the children observed, the type of target area, the type of equipment used, and the weather conditions. In addition, the conditions of the target area in relation to its accessibility, usability, supervision, development of an organised physical activity, and provision of equipment were collected. Schools were visited by the research team before the monitoring observation periods to identify and define target areas and observation points. The defined target areas and selected observation points were drawn from aerial views of schools taken from Google maps (Fig. 3). The maps were given to the observers. Same target areas and observation points were used for both observation periods. Observations were carried out in each school on two different days by two independent observers simultaneously following the SOOPEN protocol (see Supplementary data), during all recess shifts which were usually 3. The recess shifts lasted an average of 30 min, so the observations lasted a total of 1 h 30 min on average per school. Observations were collected manually on a data collection sheet that can be found in the SOOPEN protocol (see Supplementary data).

##### Health and Well-Being of Teachers

To evaluate the impact of the intervention on teachers’ thermal comfort, we selected a subsample of 80 teachers from 4 schools in the IG and 4 schools from the CG: 40 at the IG (10 per school) and 40 at the CG (10 per school). They were selected to ensure diversity (e.g. socioeconomic level, location in the neighbourhood, school size, greenery).

We monitored participants’ individual exposure to temperature and humidity during school hours using iButtonTM DS1923 Hygrochron Data Logger temperature/humidity sensors. Participants hung the iButton on the outermost layer of their clothes at chest level. The thermal discomfort index (Thom’s index, ID) was calculated by applying Thom’s formula that combines the values of relative humidity (H) and temperature (T), ID = *T* − 0.55 × (1 − 0.01*H*) × (*T* − 14.5) [43]. Measurements were carried out during a week at two time points after the implementation of interventions, November 2020 and May 2021. At the end of the monitoring week, participants completed the ASHRAE questionnaire (“Thermal environmental conditions for human occupancy”) [44] to evaluate their thermal comfort perceptions.

We also assessed the effect of the intervention on perceived school environmental quality on a subsample of 151 teachers from 21 schools (11 IG and 10 CG). We administered the validated MM040 School tool to assess the impact in terms of quality of school’s indoor and outdoor environmental conditions, and health-related problems [45]. Teachers were invited to complete the questionnaire via email sent by the school director in two assessment periods (November 2020 and May 2021). The questionnaire consisted of 50 questions to measure sociodemographic indicators, employment data, health-related habits, environmental factors in the workplace, psychosocial working conditions, former and current symptoms and health problems, and school environment.

#### Qualitative Evaluation

##### Student Perceptions of the Effects of the Interventions

We also used qualitative methods to study how the intervention affected student’s perceptions of the school environment, health and well-being, climate awareness, use of the schoolyard, physical activity, and social behaviour. We sampled a total of 59 6th-grade students (core group) from 4 schools of the IG. Schools were selected considering the following: (a) representativeness of schools with different socioeconomic levels, (b) different types of interventions, and (c) willingness of the school’s director to participate in the evaluation. In each school, a convenience sample of 10–25 volunteers were recruited to get a group with gender variability and students from different affinity groups, based on availability, willingness, and children’s motivation to participate.

The Photovoice technique [46] was used to capture students’ experience and perception regarding the interventions by using photography and written and verbal reflections about their photographs. Photovoice consisted of three sessions, which were audio-recorded and lasted from 60 to 90 min.

In the first session, participants were given digital cameras and asked to take photos in pairs considering what were the most and the least useful interventions to adapt their school to climate change effects. In the second session, students were asked to describe up to two photos and share their reflections with the group, using an adaptation of the SHOWED method [47]. Children identified the common themes of the photos based on the assigned research question. Assisted by the research team, students identified the photos that best represented the selected themes. In the last session, participants were asked about how the physical transformation of the schoolyard changed its use, social behaviour, and their feelings. They captured their opinions in a mural using words and drawings.

##### Teacher Perceptions of the Effects of the Interventions

We conducted a qualitative research to evaluate perceptions on the effects of the intervention among teachers. We included a sample of 11 teachers, one from each of the 11 IG schools. Teachers were selected from those who actively participated in the co-creation process and were also willing to participate in the focus groups.

We conducted two focus groups (five to six teachers in each group) to identify experiences and perceptions of the school’s teachers regarding the interventions. These were audio-recorded and lasted about 90 min.

Focus groups were conducted by a moderator, assisted by an observer. We used semi-structured guides covering the following topics: (1) most and least beneficial interventions for students, (2) impact of the transformations on student’s health and well-being, (3) impact of the intervention on the use of the schoolyard and social behaviour among students, (4) impact of the pedagogical process among the school community, (5) potential impact of the intervention at community level, and (6) overall satisfaction with the project.

##### User Attitudes, Experiences, and Perceptions of the Climate Shelters among Local Community

To study the use of open schoolyards as climate shelters by the local community members and their perceptions of thermal comfort and well-being, we used a spontaneous ethnography approach. The study area included the 11 IG schoolyards, which were observed during summer 2022 (non-school periods) in various sessions of 1–1.30 h (2 sessions/school). Brief semi-structured interviews were conducted to 76 users (3–5 min) and 18 caretakers (10–15 min) of the open schoolyards during the observation sessions. Participants were spontaneously selected, considering diversity in terms of gender and age.

Data were collected by audio recording and notes in a fieldwork diary. Interviews conducted to schoolyard’s users included questions related to how often they visit the schoolyard, main reasons for using it, perception of shaded areas, vegetation, and water items, favourite things about the schoolyard, and suggestions for improvement. Supervisors of the schoolyard were asked about user’s profile, main activities, and main reasons for using the schoolyard, as well as differences in schoolyard use according to heat, gender, and age. We also gather data about their perception of shaded areas, vegetation, and water items, perception of thermal comfort, their favourite things about the schoolyard, and suggestions for improvement. Both users and supervisor were asked to rate the schoolyard as a climate shelter. We collected sociodemographic information about interview participants such as gender, age, neighbourhood where they lived, and if they were part of the educational community. Observations collected information regarding user’s profile, the use of shaded areas and other spaces, interaction with green and blue measures, and social behaviour. During observations, we also gather information about the weather, shade, elements, and equipment present in the schoolyard, and quality of the interventions.

### Data Analysis

Quantitative and qualitative data will be analysed separately, and results will be converged following a concurrent mixed-method triangulation design [48]. Triangulation design is a research approach that combines quantitative and qualitative methods to provide different but complementary insights on the same topic. It is used to gain a more holistic understanding of the impact of the intervention. We aim to compare quantitative statistical results with qualitative findings by merging the separate results together in the interpretation, giving them equal weight.

#### Quantitative Data Analysis

##### Environmental Conditions and Air Quality

We will follow a pre-post-intervention evaluation strategy to compare environmental measurement trends over time. We will analyse mean and maximum daily temperature data from outdoor and indoor sensor kits for the pre-intervention summer (June–September 2020) and the post-intervention summer (June–September 2021). To account for seasonal effects, we will compute daily anomalies for each IG school smart citizen kit relative to a control kit that is unaffected by the interventions in either outdoor or indoor conditions.

To determine whether the interventions had a significant effect, we will perform a Wilcoxon signed-rank test on the daily anomalies of each intervention kit for pre- and post-intervention conditions.

To account for multiple testing errors, we will use the false discovery rate (FDR) method by Benjamini and Hochberg, setting a false discovery rate of 5%. Significant results can be interpreted as those kits whose temperature difference between the pre-intervention and post-intervention values significantly changed.

Data collected from the 1-day measurement campaigns will be used to compute kriging interpolation maps of temperature anomalies for each schoolyard kit in comparison to the control kit.

NO_2_ concentrations (µg/m^3^) will be compared with the annual average limit value established by the EU Air Quality Directive 2008/50/EC (40 µg/m^3^), and the value recommended by the World Health Organization’s (WHO) 2021 guidelines (10 µg/m^3^). We will analyse and compare NO_2_ concentrations measured at the different schools according to their location (outdoor, schoolyard, or indoor), the season of the year, and the city district where the schools are situated.

##### Health and Well-Being of Students and Teachers

To analyse data from questionnaires, tests, and systematic observations, we will calculate percentages (for categorical variables) and means or medians (for continuous variables) for pre- and post-measures. Changes between pre- and post-intervention will be assessed within groups using the McNemar test (qualitative data) and paired sample *t*-test or Wilcoxon test, as appropriate (continuous variables). We will estimate the real effect of the intervention using a difference-in-difference (DID) analytical approach, by building lineal generalised models that include the interaction term between groups (IG/CG) and time (pre/post). DID relies on the assumption of parallel trends. This is, in the absence of intervention, the unobserved differences between IG and CG are the same over time. We will assess whether our data meet such assumption. In order to do this, we will perform a comparison of the baseline measures (sociodemographic and dependent variables) between IG and CG (at individual and school level), using chi-square test (categorical data) and *t*-Student or the proper non-parametric tests (continuous variables). Models will be adjusted for sociodemographic characteristics if differences between groups are detected.

We will carry out additional sensitivity analysis to assess consistency of DID estimations, by performing complementary analysis selecting homogeneous subgroups. For instance, data will be analysed according to student’s sex and socioeconomic level of school’s neighbourhood. Different interventions were implemented according to school needs and the co-design process. Hence, stratified analyses will be also performed according to the type of intervention. All analyses will be conducted using the statistical package STATA 15.1. A *p*-value < 0.05 will be considered statistically significant.

#### Qualitative Data Analysis

Data from the Photovoice, focus groups, personal interviews, and qualitative observations will be analysed similarly. Digital audio recordings will be transcribed by multiple members of the research team and complemented with observer’s field notes. Assisted by ATLAS.ti software, a thematic analysis will be carried out through codification and categorisation processes that will be discussed and agreed to by all members of the research team. The conceptual framework we developed for this evaluation (Fig. 1) will be used to guide the selection of thematic areas. Data will be analysed incorporating the gender perspective and according to socioeconomic level of the neighbourhood whenever possible.

## Discussion

### Limitations and Strengths

The main limitation of this evaluation strategy is related to the COVID-19 pandemic. School closures prevented data collection before the intervention was implemented. Evaluation design has been adapted to include retrospective questions for pre-intervention indicators, which allows us to estimate the change, although it may lead to a recall bias. The social context and the measures implemented in the schools will most likely affect the evaluation results. However, the presence of a group for comparison helps minimise threats to internal validity. Also, integrating quantitative and qualitative data will allow us to fully capture the possible effects of the interventions.

From an environmental perspective, we were only able to monitor one pre-intervention and one post-intervention summer period due to the limited project duration, which may be considered a constraint when evaluating the outcomes.

Despite these limitations, this will be the first study to estimate the environmental and health impacts of urban interventions that aim to adapt schools to climate change effects through a combination of blue, green, and grey solutions. We will identify measures that worked and can be improved, ensuring a comprehensive assessment.

Notably, the innovation of the project also lies in the fact that we can test a diverse mix of qualitative and quantitative tools to better understand which are best suited for this type of evaluation. We adapted existing tools to meet the needs of school context and can compare low-cost and co-designed sensors with more sophisticated models to test performance and suitability.

Finally, the participatory nature of the project allows the school community to understand the challenges to be addressed and co-design the solutions, making the interventions more relevant to each context.

### Implications

Climate change is increasing the frequency, duration, and intensity of heat waves, particularly in urban areas, which adversely affect population’s health and well-being [1–5]. Schools can play a vital role in helping cities adapt to climate change and become more climate resilient. Creating more natural and comfortable school environments by implementing vegetation, water features, and shaded structures has been shown to improve physical, mental, and social well-being [14–17, 23]. Projects such as “Climate Shelters”, that aim to transform public primary schools in Barcelona into cool islands through a mix of blue, green, and grey measures, can benefit the school community and the entire neighbourhoods of Barcelona.

Given the innovative nature of the project, the evaluation will help to determine the effectiveness of the interventions to enhance school’s environmental conditions and improve well-being among students, teachers, and the whole neighbourhood. Evaluation results can contribute to the creation of recommendation guidelines for future urban planning programs to promote structural and pedagogical changes in schools. Better understanding of the effectiveness and contextual factors is also relevant to inform scale-up of interventions and to help inform the optimal use of resources.

Previous published protocols have described different designs and methods for evaluating green schoolyard initiatives [27, 28]. This protocol describes the pre-post quasi-experimental evaluation strategy design we have specifically developed to evaluate the project “Climate Shelters in schools”. This project is an innovative approach to creating more sustainable and resilient school environments, which combined greening with traditional architectural elements and water features. This study is an opportunity to identify and develop the most appropriate methods to use to evaluate this type of interventions, helping local administrations understand feasible ways to integrate monitoring and evaluation into policy and practice.

### Supplementary Information

Below is the link to the electronic supplementary material.

Supplementary file1 (PDF 658 kb)

## Data Availability

No new data were created or analysed in this study. Data sharing is not applicable to this article.
