# Supplementary material for: Adapting Schools to Climate Change with Green, Blue, and Grey Measures in Barcelona: Study Protocol of a Mixed-Method Evaluation
Source: J Urban Health. 2024 Jan 18;101(1):141–54. doi: 10.1007/s11524-023-00814-y (PMC10897086; doi:10.1007/s11524-023-00814-y)
Supplement: Supplementary file 1 — Supplementary file1 (PDF 658 kb) [file 11524_2023_814_MOESM1_ESM.pdf]

SOOPEN

System for Observing Outdoor Play Environments in Neighborhood Schools

Description and Procedures Manual

February, 2022

TABLE OF CONTENTS

PURPOSE 2

RATIONALE 2

OBSERVATION PREPARATION 3

RECORDING PROCEDURES 4

SOOPEN CODES 5

SOOPEN DEFINITIONS 7

GROUP SIZE 7

SEX 7

ACTIVITY LEVEL 7

INTERACTIONS 8

SHADE 9

ACTIVITY CODE 10

CONSIDERATIONS 11

KEY WORDS 11

REFERENCES 12

ANNEX 1. SOOPEN RECORDING FORM 13

SOOPEN
(System for Observing Outdoor Play Environments in Neighborhood Schools)

PURPOSE

SOOPEN was designed to obtain information on children's physical activity levels, group sizes, play activities, and social behavior during recess in the schoolyard, but is applicable to a wide range of structured and unstructured play settings.

RATIONALE

Children frequently engage in diverse activities and behaviors that are broadly defined as play (Ridgers et al. 2012), but little research has documented children's physical activity levels during play and how they are influenced by social contexts. Playground dynamics are multidimensional, consisting of behavioral, motivational and contextual components (Jenvey and Jenvey 2002). Play has an important role in the emotional, social, cognitive, motor, and physical development of children and helps them interact with the environment around them, learn to work in groups, resolve conflicts, develop competencies, and practice decision making skills (Ginsburg et al. 2007). Play is the most natural way for children to be active, and often has a vigorous physical activity component(Tremblay et al. 2015). Subsequently, play may increase physical activity levels in children and contribute substantially to their physical development and health. There is a need to examine factors that may influence children's physical activity in play environments, including the activities they engage in, the size of the social group they play with, gender composition of these groups, and their interactions with others (Ridgers, Stratton, and Fairclough 2006). Accordingly, these variables can be investigated in the recess schoolyard setting which offers a unique context for children to be physically active while freely engaging with same-aged peers in a variety of activities (Ridgers, Stratton, and Fairclough 2006).

A variety of tools are available for assessing physical activity and there are advantages and disadvantages of each method. Self-report tools are widely used but are limited by children's ability to accurately report details of their activity behaviors. Objective measures of physical activity (e.g., heart rate monitors, motion sensors, pedometers, and accelerometers) offer advantages over qualitative methods but are more expensive and place a burden on participants and researchers.

A method that helps to avoid limitations of both self-report measures and objective activity monitors is the direct observation technique. For example, the System for Observing Play and Leisure Activity in Youth (SOPLAY) provides objective data on the number of participants and their physical activity levels during play and leisure opportunities in targeted areas(McKenzie 2006). Separate scans are made for males and females, and simultaneous entries for contextual characteristics of areas including their accessibility, usability, and whether or not supervision, organized activities, and equipment are provided. Similarly, the System for Observing Children's Activity and Relationships during Play (SOCARP) is an objective assessment tool designed to assess children's play behavior, the size of the social groups, the type of activity, and behavior and social interaction during break periods(Ridgers, Stratton,

and McKenzie 2010). Both tools (SOPLAY and SOCARP) were considered as tools to evaluate social interactions and physical activity in school playgrounds, although to capture a global image of the dynamics developed in the whole schoolyard playground, two limitations were found when using both tools separately. SOPLAY does not assess social interactions and SOCARP, although considering behavior and social cohesion, has an individual observation methodology which does not account for group dynamics. The System for Observing Outdoor Play Environments in Neighborhood Schools (SOOPEN) proposes a combined tool to obtain data on physical activity and social interaction dynamics during recess in the schoolyard at the group level.

SOOPEN is based on momentary time sampling techniques in which systematic and periodic scans of groups of individuals and contextual factors within predetermined target areas are made. Summary counts describe the number of males and females, of a given group in a setting, and their average physical activity levels. The instrument permits physical activity level comparisons to be made among different environments or within the same environment over different time periods. Energy expenditure rates (Kcal/kg/min) can also be calculated based on previously validated constants for each level of activity (Honas et al. 2008).

OBSERVATION PREPARATION

1. Confirm with the school when and how the start and end of recess is determined. This will influence the times recorded during the observation procedure

2. Plan at least one visit to the schoolyard prior to the assessment to get a general idea of the schoolyard recess dynamics and identify Target Areas

OBSERVATION AREAS (Target Areas)

- Direct observations will be made in designated Target Areas that divide the schoolyard into scan spaces where the children are allowed to play during recess. These Target Areas will be predetermined and identified for observations prior to baseline assessments. A map will be provided to identify areas and a standard observation order established for each school. Additional target areas may be added by observers on site and then documented.

- During occasions of high student density, Target Areas will be subdivided into smaller Scan Spaces so that accurate measures can be obtained. Observers will use schoolyard playground markings and permanent structures to help determine appropriate Scan Spaces within each Target Area. Data from these smaller spaces will be aggregated to provide an overall measure for each Target Area. NOTE: A decision to subdivide a Target Area depends upon the (1) number of students in the area and (2) the type of student activity. Fast moving activities with students clustered

together and moving in diverse directions (e.g., during soccer and basketball) may require smaller scan spaces.

3. Prior to arrival at the school, prepare observation materials including: mobile phone* and stopwatch clipboard, sufficient SOOPEN recording forms, and pencils.

*Mobile phone is used as a timer during the observation. It also serves to record the start and finish times and to check the weather and temperature (the same weather app should be used by all the observers)

4. Arrive at the school at least 30 minutes prior to the official start of data collection. Review the sequence for observing Target Areas. Visit each Target Area in order and plan how to subdivide it into Scan Spaces if necessary. Prepare mentally by scanning each area a few times. If feasible, take pictures of each Target Area in order to remember them for the subsequent assessments, data analysis and interpretation of results. Take into account that children should not appear in photos without prior informed consent.

RECORDING PROCEDURES

The objective is to obtain an accurate measure of play and social activity during unstructured time on the schoolyard. Each school could have specific recess time and they could be applying contingency plans for exceptional situations, such as during the COVID-19 pandemic. Taking into consideration the previously mentioned, divide the recess time among the identified Target Areas, enabling enough time to change observation position among Scan Areas if needed. For example, in a schoolyard with a recess time of 30 minutes, with 3 Target Areas, 7 minutes scan rotation could be performed for each Target Area. Start at Target Area 1 at the beginning of the recess; then walk directly to Target Area 2 and subsequent Target Areas in a designated rotation. If there are different recess schedules for different grades, it is recommended observing the scan area as many times as there are different turns. If the Target Area is subdivided into smaller scan spaces, proportional time fraction should be assigned to each scan space along the total minutes of scan time.

For each Target Area:

1. Place yourself in a position to observe the target Area at least 5 minutes before the recess starts. Try to be as unnoticed as possible and do not interfere with any recess activities.

2. Wait a few minutes to start the observation in order to let the children take up the space and start activities. Children will most likely need some time to settle in the schoolyard, take some equipment or organize games.

3. On the observation form (Annex 1), enter the **Date**, the **School ID**, **Observer ID**, if it was a **Reliability** assessment, the **Weather**, and the **Temperature**.

4. Enter the **Target Area/Scan Area** and the **Start Time** for each scan, and the grade of children you are going to observe (if known). Record the contextual variables for each area (see SOOPEN codes).

5. Scan each entire Target Area for the predefined time. Always scan from LEFT to RIGHT and from FRONT to BACK. Observe each group in the area once. Prior to starting to fill up the codes of the recording form for each observed group, take a little time to identify the activity in order to be able to describe it. Record the contextual variables for each area (see SOOPEN codes).

6. If any children change the group and start a new activity, record them as a new group, even if any of the children were previously recorded in another group. If a group that has been already recorded moves to another scan area but they continue doing the same activity with the same members, do not record them again. If the group size changes in a different area, record as a different group.

7. Do NOT BACK-TRACK to count new children entering into the scan area.

SOOPEN CODES

Date Enter the date (dd/mm/yyyy) of the observation

School ID Enter the school ID

Observer Enter your observer ID code

Reliability Circle **NO** unless you are the second observer and your data will serve as a reliability measure

Area Refers to the number of a previously designated School Target Area (see school map). If necessary, add an additional area, describe it, and give it a number.

Type of area Add a brief description of the Target Area such as, basketball or football pitch, sandbox, picnic/gathering area, or garden.

Start Time Enter the start time (24:00 hours) of the sweep for that designated area.

End Time Enter the end time (24:00 hours) of the sweep for that designated area

Grade Enter the grade of children you are going to observe:

- **Preschool education** (3 - 5 years old)
- **1st cycle primary school**: 1st and 2nd grade (6-7 years old)
- **2nd cycle primary school**: 3rd and 4th grade (8-9 years old)
- **3rd cycle primary school**: 5th and 6th grade (10-11 years old)

Condition Circle **No** or **Yes** to describe specific conditions for each designated observation area. If a Target Area is inaccessible (A = No), do not code the other four conditions.

A = Area is accessible (e.g., not locked or rented to others)
U = Area is usable for activity (e.g., is not excessively wet or windy)
S = Area is supervised by designated school or adjunct personnel (e.g., teachers, playground supervisors, volunteers). The supervisor must be in or adjacent to that specific area (i.e., available to direct students and respond to emergencies), but does not have to be instructing, officiating, or organizing activities.

Number of supervisors: Record the number of children's supervisors during the recess.

OPA = Organized Physical Activity (i.e., scheduled, with leadership by school or agency personnel apparent) is occurring in the area (e.g., intramurals, interscholastic practices, fitness stations)

E = Equipment provided by the school or other agency is present (e.g., balls, jump ropes). Code **No** if the only equipment is permanent (e.g., basketball hoops) or is owned by students themselves.

Type of equipment: If appropriate, write which type of equipment is provided as additional descriptive information.

Weather Select the appropriate pictogram according to the actual weather

- Sunny 0% of cloud cover
- Partly sunny 25-50% of cloud cover
- Middle cloudy 50-75% of cloud cover
- Totally cloudy 100% of cloud cover

- Showers 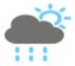
- Rain 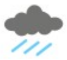
- Windy 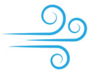

Temperature Enter Celsius temperature at the start of the observation period.

SOOPEN DEFINITIONS

Group size

The social group size reflects the number of children playing/interacting. Code the group size (**A, S, M, L**) at the record prompt.

NOTE: Do not include groups of adults (e.g. teachers, supporting staff, organizing, refereeing) in the counting (a comment should be added). If a child is interacting with an adult, they can be counted as a small group (2 people).

Alone (**A**)	A child is alone, and not interacting with any other person.
Small (**S**)	Group of 2 to 4 people.
Medium (**M**)	Group of 5 to 9 people.
Large (**L**)	Group of 10 or more people.

When a child is in transition between groups of different sizes on the record prompt, consider him/her only as part of the first group observed. For example, count and code L (large) if the child is just leaving a group of 10 children and walking away on his/her own.

Sex

For each group observed, count and register the <u>number</u> of **Girls** and **Boys**.

Activity level

Code the activity level/body position of the majority of children within the group into one of the 5 following categories using momentary time sampling.

1. Lying
2. Sitting
3. Standing
4. Walking/moderate
5. Very active/vigorous

Pictograms corresponding to codes 1-5 are facilitated in this category of the recording form to make the register easier. .

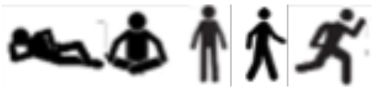

Code **1-4** unless children are expending more energy than is required for an ordinary walk.

Code **5** (very active) for any activity that requires children to expend more energy than they would use for an ordinary walk. Examples include running, jogging, hopping, stretching, wrestling with a peer, and fast movements on the spot.

When activity levels of children in the group correspond to more than one category, circle the category that represents the majority of children. For example, if in a medium group (M) formed by 5 children, 2 of them are standing while the rest are sitting, code level **2** (sitting).

Other sample codes:
kneeling (weight on knees only) = 3
kneeling (weight on knees and buttocks) = 2
inactive "on all fours" = 3
seated swinging (arms producing no momentum) = 2
seated swinging (arms producing momentum) = 4
carrying, pushing, pulling objects (obviously struggling) = 5

Interactions
Child social behavior and interactions are recorded under the Interactions variable. Interactions are divided into two main categories: **prosocial** and **antisocial**. Each of these categories is further classified into a **(a) physical** or **(b) non-physical** response category.

A physical interaction typically involves physical contact or physical engagement in a physical activity. A nonphysical interaction has no physical contact (McKenzie 2006).

Pro-social behaviors involve positive social interactions with other children. These include behaviors in which the child interacts pleasantly with another child by providing encouragement, support, assistance, feedback, or explanation (e.g., good sporting or sportsmanship behaviors).

a) Physical pro-social behaviors include assisting injured children; helping others get up; spotting, retrieving objects or equipment for others; holding hands, linking arms, or gently hugging another child; and giving a "high five." Fantasy play/imaginative play that is characterized by physical contact should be coded here. Also, if the child is alone at the moment of the observation but involved in a sport activity/active game (e.g., goalkeeper)

b) Non-physical pro-social behaviors include (a) verbal behaviors such as consoling the injured; providing rules or instruction; supervising and organizing games; offering thanks; offering to equalize competition; praising others or approving their behavior; and (b) nonverbal behaviors such as giving a thumbs-up gesture or victory sign, clapping for a team-mate or opponent during a game.

Antisocial behaviors involve negative social interactions with other children. These include a child interacting with another child in such a manner that intimidation, provocation, animosity, or disapproval are conveyed in the tone and content of the interaction (e.g., bullying).

a) Physical antisocial behaviors include hitting, kicking, grabbing, shoving, pushing, punching, and taking equipment away from others. It also includes non-contact, but threatening or negative behaviors, such as when a child uses his/her body to disrupt games or the activities of others. Do not code strategies that are a traditional part of games as negative social behaviors (e.g., blocking out or shielding during soccer or basketball, catching someone during a chasing game).

b) Non-Physical antisocial behaviors include name calling, teasing, arguing, ridiculing, swearing, making sarcastic comments, obscene gestures, and verbally refusing to let children enter into games.

Protocol for Coding Interactions

During the observation, code the main social interaction (N, PS, VS, PC, VC) that each observed group was engaged in. Record the type of interaction that represents the majority of the group. Use the following five codes to indicate if the target group engaged in:

None (**N**)	No pro- or antisocial behaviors
Physical sportsmanship (**PS**)	Physical pro-social behavior during the interval.
Verbal sportsmanship (**VS**)	Non-physical pro-social behavior during the interval.
Physical conflict (**PC**)	Physical antisocial behavior during the interval.
Verbal conflict (**VC**)	Non-physical antisocial behavior during the interval.

Shade
Record the use of spaces with physical covers, such as arbor, trees, porch, awning of each observed group (Maddock et al. 2007).

None (**N**)	All the members of the group are uncovered and potentially or completely exposed to full sun

Partial (**P**)	The group is partially covered. The area is partially shaded.
Fully covered (**FC**)	All the members of the group are covered.

Activity code
Enter the activity code (or name) of each activity observed in the group.

1. Football
2. Basketball
3. Other sports*
4. Active games**
5. Running (not involved in sport / active game)
6. Walking (not involved in sport / active game)
7. Talking
8. Eating (e.g., eating lunch outside, eating tuck) / drinking
9. Reading, writing, artwork
10. Sandbox, playing with plants
11. Playing board games (e.g., chess, draughts, noughts and crosses)
12. Fantasy play/Imaginative play
13. Viewing others' games (i.e., as a spectator, not as a participant)
14. Using computer consoles/mobile phones (if allowed)
15. Other activities

* Children are engaged in sport-related activities. This activity can be a modification of the sport, without its official structure (e.g. rules, numbers of players). Examples include:

Rugby, field hockey
Tennis, badminton
Rounders, baseball, softball, cricket, climbing, ping-pong
Weight lifting, stretching, acrobatics, gymnastics, frisbee.

** Children are engaged in organized physical activity, but a non-sport. Examples of active games include:

Chasing games/tag/tick/manhunt/hide and seek
Dodgeball, four-square
Racing
Hopscotch
Play fighting/fantasy play/imaginative play
Rough and tumble (includes wrestling and grappling)
Throwing and catching games (e.g., king ball, end zone)
Verbal games that involve actions (e.g., ring-a-roses, farmer's in his den)
Target games (e.g., bullseye, hoop ball)
Clapping games (e.g., a sailor went to sea, short legged sailor)

Hula Hoop, skipping rope, dancing.

Comments - Describe any events or features that may help explain any of the above data.

Categories for activity types:
Each activity type listed above belongs to one of this four activity types (in order to clarify the classification; these categories should not be used at the record prompt):

Sports (SP): Activity codes **1-3**. Children are engaged in sport-related activities. This activity can be a modification of the sport, without its official structure (e.g., rules, numbers of players).

Active Games (G): Activity code **4**. Children are engaged in a physically active game.

Locomotion (L): Activity code **5-6**. Children are engaged in a locomotor activity (e.g., in transition) that was NOT part of a sport or active game.

Sedentary (S): Activity codes **7-15**. Children are engaged in a sedentary activity.

CONSIDERATIONS

Fieldwork diary

After the observation, we recommend writing a brief description about what you have observed.

KEY WORDS

Target Area - A predetermined observation area in which students may potentially engage in physical activity. A number of Target Areas will be established for each school.

Scan Space - A subdivision of a Target Area in which the assessor makes an observation scan. Target Areas are subdivided into Scan Spaces when the number of students is large and they are engaged actively.

Scan - A single observation movement from left to right across a Target Area or Scan space. During a sweep, each group of children in the area is counted and coded.

REFERENCES

Ginsburg, Kenneth R. et al. 2007. "The Importance of Play in Promoting Healthy Child Development and Maintaining Strong Parent-Child Bonds." *Pediatrics* 119(1): 182–91. https://pubmed.ncbi.nlm.nih.gov/17200287/ (February 13, 2022).

Honas, Jeffery J. et al. 2008. "The System for Observing Fitness Instruction Time (SOFIT) as a Measure of Energy Expenditure During Classroom-Based Physical Activity." *Pediatric exercise science* 20(4): 439. /pmc/articles/PMC3711688/ (February 6, 2022).

Jenvey, Vickii B., and Heidi L. Jenvey. 2002. "Criteria Used to Categorize Children's Play: Preliminary Findings." *Social Behavior and Personality* 30(8): 733–40.

Maddock, Jay E., David L. O'Riordan, Kevin B. Lunde, and Alana Steffen. 2007. "Sun Protection Practices of Beachgoers Using a Reliable Observational Measure." *Annals of behavioral medicine : a publication of the Society of Behavioral Medicine* 34(1): 100–103. https://pubmed.ncbi.nlm.nih.gov/17688402/ (February 13, 2022).

McKenzie, Thomas L. 2006. *SOPLAY: System for Observing Play and Leisure Activity in Youth*.

Ridgers, Nicola D. et al. 2012. "Physical Activity During School Recess: A Systematic Review." *American Journal of Preventive Medicine* 43(3): 320–28.

Ridgers, Nicola D., Gareth Stratton, and Stuart J. Fairclough. 2006. "Physical Activity Levels of Children during School Playtime." *Sports Medicine 2006 36:4* 36(4): 359–71. https://link.springer.com/article/10.2165/00007256-200636040-00005 (November 14, 2021).

Ridgers, Nicola D., Gareth Stratton, and Thomas L. McKenzie. 2010. "Reliability and Validity of the System for Observing Children's Activity and Relationships During Play (SOCARP)." *Journal of Physical Activity and Health* 7(1): 17–25. https://journals.humankinetics.com/view/journals/jpah/7/1/article-p17.xml (November 7, 2021).

Tremblay, Mark S. et al. 2015. "Position Statement on Active Outdoor Play." *International Journal of Environmental Research and Public Health* 12(6): 6475. /pmc/articles/PMC4483712/ (February 13, 2022).

ANNEX 1. SOOPEN Recording form

Page n. ___ - SIDE A

ANNEX 1

SOOPEN Recording Form

Date: __/__/____ Observer:

School: Reliability: No Yes

Recess time start: __:__

Recess time end: __:___

Area: _______ Subarea:______

Type of area: ________________

Grade: _______

A: No Yes

U: No Yes

S: No Yes Nº of supervisors: __

OPA: No Yes

E: No Yes

Type of E: __________________

Area: _______ Subarea:______

Type of area: ________________

Grade: _______

A: No Yes

U: No Yes

S: No Yes Nº of supervisors: __

OPA: No Yes

E: No Yes

Type of E: __________________

WEATHER

Sunny

Cloudy

Showers

Rain

Windy

TEMPERATURE __°C

Area / Subarea	Start Time	End Time	GROUP SIZE 1 2-4 5-9 10+	SEX		ACTIVITY LEVEL	INTERACTIONS	SHADE	ACTIVITY CODE	COMMENTS
				Girls	Boys					
			A S M L				N PS VS PC VC	N P FC		
			A S M L				N PS VS PC VC	N P FC		
			A S M L				N PS VS PC VC	N P FC		
			A S M L				N PS VS PC VC	N P FC		
			A S M L				N PS VS PC VC	N P FC		
			A S M L				N PS VS PC VC	N P FC		
			A S M L				N PS VS PC VC	N P FC		
			A S M L				N PS VS PC VC	N P FC		
			A S M L				N PS VS PC VC	N P FC		
			A S M L				N PS VS PC VC	N P FC		
			A S M L				N PS VS PC VC	N P FC		
			A S M L				N PS VS PC VC	N P FC		
			A S M L				N PS VS PC VC	N P FC		
			A S M L				N PS VS PC VC	N P FC		

Activity Codes:

1. Football; **2.** Basketball; **3.** Other sports; **4.** Active games; **5.** Running; **6**. Walking; **7.** Talking; **8.** Eating; **9.** Reading, writing, artwork; **10.** Sandbox; **11.** Playing board games; **12**. Fantasy play; **13.** Viewing others' games; **14.** Using computer consoles/mobile phones; **15.** Other activities

SIDE B

Area / Subarea	Start Time	End Time	GROUP SIZE<br>1 2-4 5-9 10+	SEX<br>Girls	 Boys	ACTIVITY LEVEL	INTERACTIONS	SHADE	ACTIVITY CODE	COMMENTS
			A S M L				N PS VS PC VC	N P FC		
			A S M L				N PS VS PC VC	N P FC		
			A S M L				N PS VS PC VC	N P FC		
			A S M L				N PS VS PC VC	N P FC		
			A S M L				N PS VS PC VC	N P FC		
			A S M L				N PS VS PC VC	N P FC		
			A S M L				N PS VS PC VC	N P FC		
			A S M L				N PS VS PC VC	N P FC		
			A S M L				N PS VS PC VC	N P FC		
			A S M L				N PS VS PC VC	N P FC		
			A S M L				N PS VS PC VC	N P FC		
			A S M L				N PS VS PC VC	N P FC		
			A S M L				N PS VS PC VC	N P FC		
			A S M L				N PS VS PC VC	N P FC		
			A S M L				N PS VS PC VC	N P FC		
			A S M L				N PS VS PC VC	N P FC		
			A S M L				N PS VS PC VC	N P FC		
			A S M L				N PS VS PC VC	N P FC		
			A S M L				N PS VS PC VC	N P FC		

Activity Codes:

1. Football; **2.** Basketball; **3.** Other sports; **4.** Active games; **5.** Running; **6**. Walking; **7.** Talking; **8.** Eating; **9.** Reading, writing, artwork; **10.** Sandbox; **11.** Playing board games; **12**. Fantasy play; **13.** Viewing others' games; **14.** Using computer consoles/mobile phones; **15.** Other activities
